# Comparative toxicogenomic responses of mercuric and methyl-mercury

**DOI:** 10.1186/1471-2164-14-698

**Published:** 2013-10-11

**Authors:** Matthew K McElwee, Lindsey A Ho, Jeff W Chou, Marjolein V Smith, Jonathan H Freedman

**Affiliations:** 1 Laboratory of Toxicology and Pharmacology, National Institute of Environmental Health Sciences, NIH, 111 T.W Alexander Drive, Research Triangle Park, P.O. Box 12233, 27709 Durham, NC, USA; 2 SRA International, Inc., Durham, NC, USA; 3 Microarray and Genome Informatics Group, National Institute of Environmental Health Sciences, NIH, Research Triangle Park, Durham, NC, USA; 4 Current address: Department of Biostatistical Sciences, Wake Forest University School of Medicine, Medical Center Boulevard, 27157 Winston-Salem, NC, USA

**Keywords:** *C. elegans*, Inorganic mercury, Organic mercury, Methylmercury, Transcriptome, Metal toxicity

## Abstract

**Background:**

Mercury is a ubiquitous environmental toxicant that exists in multiple chemical forms. A paucity of information exists regarding the differences or similarities by which different mercurials act at the molecular level.

**Results:**

Transcriptomes of mixed-stage *C. elegans* following equitoxic sub-, low- and high-toxicity exposures to inorganic mercuric chloride (HgCl_2_) and organic methylmercury chloride (MeHgCl) were analyzed. In *C. elegans*, the mercurials had highly different effects on transcription, with MeHgCl affecting the expression of significantly more genes than HgCl_2_. Bioinformatics analysis indicated that inorganic and organic mercurials affected different biological processes. RNAi identified 18 genes that were important in *C. elegans* response to mercurial exposure, although only two of these genes responded to both mercurials. To determine if the responses observed in *C. elegans* were evolutionarily conserved, the two mercurials were investigated in human neuroblastoma (SK-N-SH), hepatocellular carcinoma (HepG2) and embryonic kidney (HEK293) cells. The human homologs of the affected *C. elegans* genes were then used to test the effects on gene expression and cell viability after using siRNA during HgCl_2_ and MeHgCl exposure. As was observed with *C. elegans*, exposure to the HgCl_2_ and MeHgCl had different effects on gene expression, and different genes were important in the cellular response to the two mercurials.

**Conclusions:**

These results suggest that, contrary to previous reports, inorganic and organic mercurials have different mechanisms of toxicity. The two mercurials induced disparate effects on gene expression, and different genes were important in protecting the organism from mercurial toxicity.

## Background

Mercury is a ubiquitous environmental contaminant that exists as either an organic or inorganic species that undergoes complex cycling in the environment and *in vivo*. Humans are exposed to various forms of inorganic mercury including elemental mercury (Hg^0^), mercury salts and ionic mercury (Hg^+^ or Hg^2+^)*.* Elemental mercury has long been used as a principal component of dental amalgams, resulting in the exposure of individuals wearing amalgams and dental professionals to mercury vapor. Other occupational exposures to mercury vapor include workers in chloralkali plants, fluorescent lamp factories and artisanal gold mines [1,2]. Exposure to mercury vapor can result in tremors; deficits in information processing speed, psychomotor speed and manual dexterity; psychological disturbances; and has been associated with Alzheimer’s disease [3,4]. The kidney is also a primary site of accumulation and toxicity of inorganic mercury. Environmental exposure to inorganic mercury is associated with an increase in mortality from kidney disease [2,5].

Inorganic mercury from natural and anthropogenic sources is converted to methylmercury. Methylmercury biomagnifies, so that animals at the top of the food chain have methylmercury levels that are orders of magnitude higher than those at the bottom [6]. Humans are exposed to methylmercury through fish consumption. It is estimated that 7% of U.S. women of childbearing age have levels of methylmercury that exceed the U.S. EPA reference dose of 0.1 μg/kg body weight/day [7]. Methylmercury poisoning outbreaks in the 1950s and 1960s in the area surrounding Minamata Bay in Japan resulted in paresthesia, ataxia, loss of vision, and death in adults [8]. What was particularly striking, however, was the sensitivity of the *in utero* life stage to methylmercury exposure. Mothers with no overt toxicity gave birth to children with gross cognitive and anatomical defects [9]. A large epidemiological study investigating the effects of prenatal mercury exposure in populations that consume large amounts of seafood found a significant correlation between mothers’ mercury levels during gestation and cognitive deficits in children [10,11].

Previous research suggested that the inorganic mercurial, HgCl_2_, and the organic mercurial, methylmercury chloride (MeHgCl), had similar mechanisms of toxicity. It has been hypothesized that organic mercury is converted to the inorganic species and that the latter is the active form of the metal. Both HgCl_2_ and MeHgCl cause oxidative stress [12,13]. It is believed that oxidative stress is caused by the depletion of glutathione and other antioxidants, since neither mercurial is redox active *in vivo*[14]. Other proposed mechanisms of HgCl_2_ and MeHgCl toxicity include microtubule disruption [15,16], inhibition of mitochondrial function, and disruption of intracellular calcium levels [17-20]. Microarray studies examining the effects of HgCl_2_ or MeHgCl on gene expression found differential expression of genes involved in the oxidative stress response, protein degradation, mitochondrial dysfunction, endoplasmic reticulum stress and phase II metabolism [21-23].

The results of previous toxicity studies suggest that HgCl_2_ and MeHgCl act via similar mechanisms [24]. The few studies that have directly compared the effects of inorganic and methylmercury also suggest that the two mercurials have similar mechanisms of action. Freitas, *et al*. found that both inorganic and methylmercury inhibit Ca^2+^-ATPase and disrupted Ca^2+^ transport in brain microsomes [25]. Both mercurials stimulated release of noradrenaline from rat hippocampal slices and had similar effects on inflammatory cytokine release in lipopolysaccharide-stimulated peripheral blood mononuclear cells [26,27].

In the present study, whole-genome microarrays were used to assess the effects of sub-, low- and high-toxicity concentrations of HgCl_2_ and MeHgCl on the *C. elegans* transcriptome. To define the genes that are critical in the *C. elegans* response to mercurial exposure, RNA interference (RNAi) was used to assess the effect of gene knockdown on *C. elegans* growth during mercurial exposure. Of the 599 genes tested, decreased expression of 18 genes significantly affected *C. elegans* growth in response to either mercurial. Only two of these, however, significantly impacted growth during both HgCl_2_ and MeHgCl exposures. The effects of HgCl_2_ and MeHgCl on the steady-state mRNA levels of nine human homologs of *C. elegans* genes critical in the mercurial response were determined in human neuroblastoma (SK-N-SH), hepatocellular carcinoma (HepG2), and embryonic kidney (HEK293) cells. As was observed in *C. elegans*, HgCl_2_ and MeHgCl produced unique responses on gene expression and different genes were critical in the cellular response. The current results demonstrate that inorganic and methylmercury differentially affect gene expression and that different genes are critical in the cellular response to the two mercurials. This suggests that, contrary to previous reports, inorganic and organic mercurials have unique mechanisms of action.

## Results

### Genes differentially expressed in response to mercurial exposure

The effects of mercurials on gene expression in *C. elegans* were assessed after exposure to sub-, low- and high-toxicity concentrations of HgCl_2_ and MeHgCl. Sub-, low- and high-toxicity concentrations were determined based on a previous study that compared the toxicity of HgCl_2_ and MeHgCl on *C. elegans* growth, reproduction, feeding, and locomotion [28]. The effects of mercurials on the steady-state mRNA levels of the *C. elegans* stress-response genes; *gcs-1* (γ-glutamylcysteine synthetase), *gst-38* (glutathione *S*-transferase), and heat shock protein genes *hsp-16.2* and *hsp-*70; were also assessed [28]. The population distribution of 7,000 nematodes was determined for each mercurial exposure prior to RNA isolation. No treatment groups had a population distribution different from untreated control. This ensured that differences in gene expression were not the result of changes in the number of *C. elegans* at any individual life stage (Additional file 1: Figure S3).

A total of 3,207 genes were significantly, differentially expressed among the six exposure conditions (fold-change ≥ 2, p < 0.01). Exposure to increasing concentrations of both HgCl_2_ and MeHgCl resulted in increasing numbers of differentially expressed genes (DEGs). At each level of toxicity, however, MeHgCl exposure produced a greater number of DEGs (Table 1).

**Table 1:** **Effects of Mercurials on Gene Expression in *C. elegans***

**Toxicity**	**HgCl**_**2**_		**MeHgCl**	
	**Concentration (μM)**	**Gene number**	**Concentration (μM)**	**Gene number**
sub	2.0	8	0.75	44
		(8 ↑, 0 ↓)		(35 ↑, 9 ↓)
low	7.5	74	2.0	419
		(68 ↑, 6 ↓)		(247 ↑, 172 ↓)
high	20	403	7.5	2,791
		(316 ↑, 87 ↓)		(1,604 ↑, 1,187 ↓)

Number of genes showing increased (↑) or decreased (↓) steady state mRNA levels.

**Figure 1:**
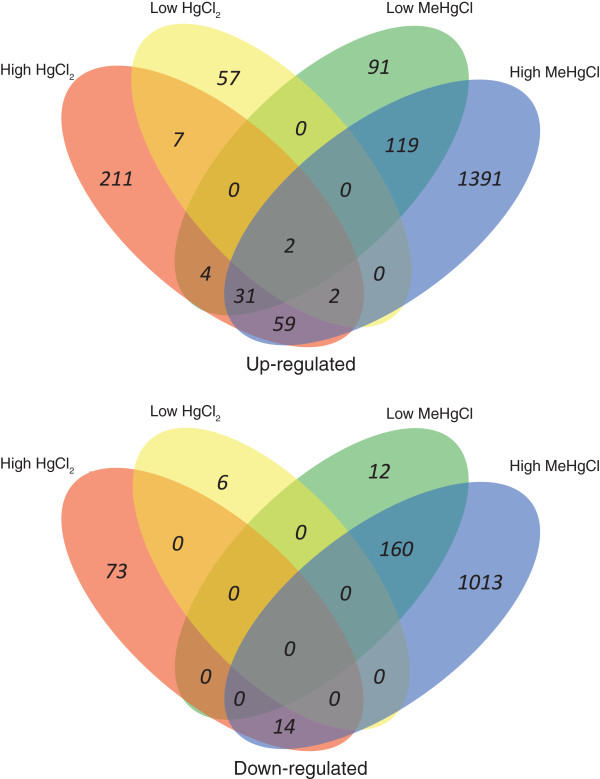
**Venn diagrams of up-regulated and down-regulated genes following low- and high-toxicity HgCl**_**2 **_**and MeHgCl exposures.** Venn diagram indicating which genes are commonly and uniquely up- or down-regulated between the low- and high-toxicity HgCl_2_ and MeHgCl exposures.

**Figure 2:**
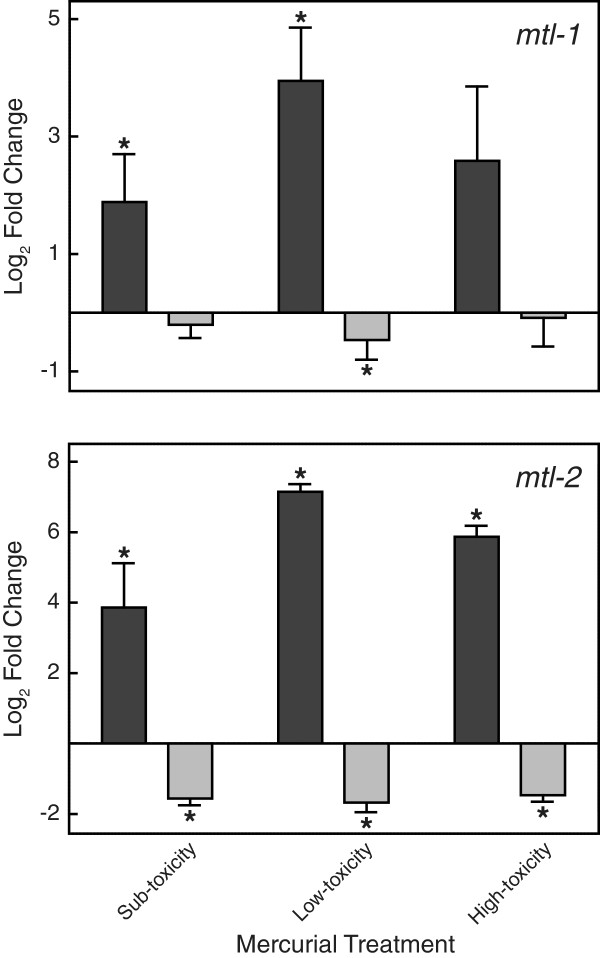
**Effects of HgCl**_**2 **_**and MeHgCl on *C. elegans *metallothionein expression.** Wild-type nematodes were exposed to sub-toxicity (2.0 μM for HgCl_2_; 0.75 μM for MeHgCl ), low-toxicity (7.5 μM for HgCl_2_; 2.0 μM for MeHgCl), and high-toxicity (20 μM for HgCl_2_; 7.5 μM for MeHgCl) mercurial concentrations for 24 h. *Dark bars*, HgCl_2_; *light bars*, MeHgCl. Steady-state levels of *mtl-1* and *mtl-2* were measured using qRT-PCR. Results are displayed as mean log_2_ ± SEM. Significant differences (p < 0.05) relative to untreated *C. elegans* are designated with an asterisk.

The five genes that had the largest increase or decrease in expression for each treatment condition are presented in Table 2. All DEGs are presented in Additional file 2: Table S4. Figure 1 shows the similarity among commonly up- and down-regulated genes at low- and high-toxicity mercurial exposures. Very few genes were similarly affected by both HgCl_2_ and MeHgCl exposures. The only genes whose expression was affected at low- and high-toxicity exposures to both mercurials were *ugt-21*, UDP-glucuronosyl transferase, and C15B12.8, an uncharacterized gene with high similarity to sarcosine oxidase. There were 24 up-regulated and eight down-regulated genes after exposure to each of the three MeHgCl concentrations (Table 3). Among the three HgCl_2_ exposures the only up-regulated gene was metallothionein-2 (*mtl-2*). Conversely, *mtl-2* was down-regulated in response to all MeHgCl exposures. qRT-PCR confirmed these microarray results, showing that *mtl-2* was up-regulated by all HgCl_2_ treatments and down-regulated by MeHgCl. Similarly, *mtl-1* was up-regulated at sub- and low-toxicity HgCl_2_ exposures, and down-regulated in low-toxicity MeHgCl exposure (Figure 2).

**Table 2:** Most significantly affected mercurial-responsive genes

**MeHgCl**								
**Sequence name**	**Gene name**	**Fold change**	**Sequence name**	**Gene name**	**Fold change**	**Sequence name**	**Gene name**	**Fold change**
**0.75 μM**			**2 μM**			**7.5 μM**		
Y32G9A.1	*gst-37*	9.4	Y32G9A.1	*gst-37*	77	Y32G9A.1	*gst-37*	100
Y1H11.2	*gst-35*	8.5	F11A5.12	*stdh-2*	24	F11A5.12	*stdh-2*	98
C29F3.1	*ech-1*	4.2	Y1H11.2	*gst-35*	22	W08E12.2		69
F35E12.5		4.0	H23L24.5	*pme-4*	22	F22E5.6		68
M199.7		4.0	Y43F8C.1	*nlp-25*	19	Y53F4B.35	*gst-31*	67
F37B1.8	*gst-19*	-4.2	T26H2.5		-11	C13A2.4		-99
T08G5.10	*mtl-2*	-3.2	Y32B12A.1		-10	F46B3.17	*col-163*	-97
T26H2.5		-3.1	R05D8.11		-7.8	R05D8.11		-93
C05E4.14	*srh-2*	-2.2	F37B1.8	*gst-19*	-5.9	F53F4.7		-91
ZK666.6	*clec-160*	-2.1	E03H12.3	*clec-176*	-5.4	T26H2.5		-88
**HgCl**_**2**_								
**2 μM**			**7.5 μM**			**20 μM**		
T08G5.10	*mtl-2*	3.1	T08G5.10	*mtl-2*	42	F22E10.4	*pgp-15*	84
K01D12.1		2.1	T08G5.1		29	C15B12.8		43
M02D8.4	*asns-2*	2.1	K11G9.6	*mtl-1*	12	C45B2.3		25
F07C4.10		2.1	F56A4.2		11	C17H1.8		21
F15H10.8		2.1	Y39B6A.1		7.9	Y70C5C.2	*clec-9*	21
			T25D10.2		-3.8	K03B8.11		-6.0
			F49H6.12		-3.7	T15B7.3	*col-143*	-5.2
			F29A7.7	*clec-20*	-3.7	Y57A10C.1		-5.2
			F14F8.4	*srz-103*	-2.9	K05F6.4		-5.2
			C43G2.2	*bicd-1*	-2.8	F54E7.5	*sdz-21*	-5.1

**Table 3:** **Differentially expressed *C. elegans *** **genes following MeHgCl exposures**

**Sequence name**	**Gene name**	**MeHgCl concentration (μM)**		
		**0.75**	**2.0**	**7.5**
B0507.8		2.3	2.2	2.0
B0554.6	*dod-20*	2.3	3.3	8.5
C15B12.8		2.7	14	14
C33A12.6	*ugt-21*	2.1	3.7	3.5
C34H4.1		2.6	6.6	13
F07E5.9		2.3	4.3	4.7
F11A5.12	*stdh-2*	3.8	24	98
F11D11.3		2.5	6.5	33
F15B9.1	*far-3*	2.2	3.8	6.3
F35E12.5		4.0	17	32
F37B1.2	*gst-12*	2.1	7.0	39
F53B2.2	*tsp-4*	2.2	5.3	15
F56D5.3		2.2	5.0	7.0
K08F4.7	*gst-4*	2.4	4.4	10
M199.7		4.0	4.9	4.1
T04H1.9	*tbb-6*	2.4	9.6	36
W06H8.2		2.9	9.9	29
Y1H11.2	*gst-35*	2.9	9.9	29
Y32G9A.1	*gst-37*	9.4	77	100
Y39G10AR.6	*ugt-31*	2.1	4.4	5.7
Y43F8C.1	*nlp-25*	3.5	19	17
Y45F10B.1	*tsp-5*	2.1	5.4	17
ZC239.14		2.5	4.4	11
ZK697.6	*gst-21*	2.9	4.2	2.6
C05E4.14	*srh-2*	-2.2	-3.4	-4.5
C11E4.7		-2.1	-3.4	-6.9
C15A11.7		-2.1	-3.9	-6.7
F11A5.9		-2.1	-2.8	-8.3
F37B1.8	*gst-19*	-4.2	-5.9	-5.4
T08G5.10	*mtl-2*	-3.2	-3.5	-3.5
T26H2.5		-3.1	-11	-88
ZK666.6	*clec-60*	-2.1	-3.2	-3.0

**Figure 3:**
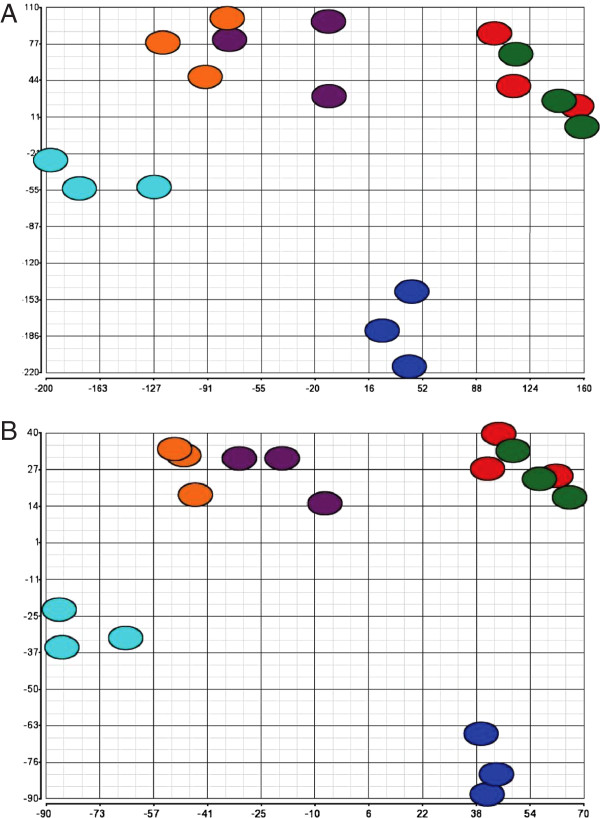
**Principal components analysis.** Panel **A**, PCA using all of the genes on the microarray; Panel **B**, PCA using only significantly, differentially expressed genes. Analyses were performed on three pairs of independent biological replicates. Treatments are designated: 2.0 μM (red), 7.5 μM (green) and 20 μM (dark blue) for HgCl_2_ and 0.75 μM (purple), 2.0 μM (orange) and 7.5 μM (light blue) for MeHgCl.

Principal components analysis (PCA) and hierarchical clustering were performed to determine the reproducibility of the mercurial-induced changes in the transcriptome, as well as visualize global effects of HgCl_2_ and MeHgCl on *C. elegans* gene expression. PCA with all genes showed tight spatial positioning of replicates indicating high experimental reproducibility (Figure 3A). The first principal component, which accounted for 33% of the variation in the data, segregated by mercurial treatments, while the second principal component, which accounted for 22% of the variation, segregated by toxicity treatments or concentration. PCA using only differentially expressed genes yielded similar results, but the first two principal components accounted for 85% of the variability (Figure 3B).

**Figure 4:**
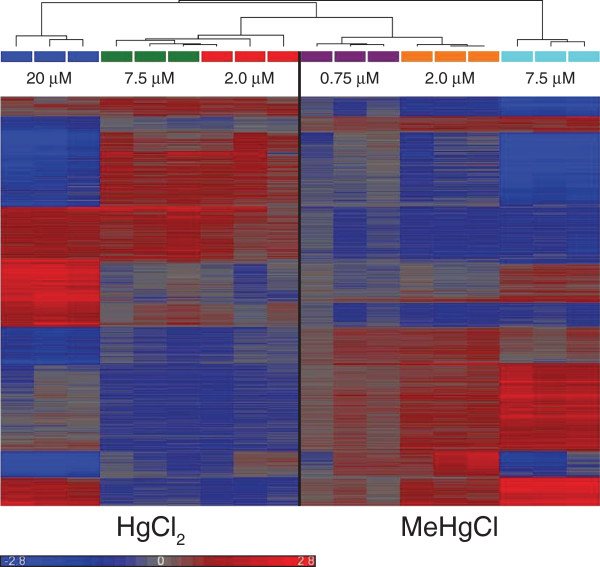
**Hierarchical clustering of microarray experimental replicates.** Hierarchical clustering was performed using three independent biological replicates for *C. elegans* exposed to sub-, low- and high-toxicity HgCl_2_ and MeHgCl concentrations. Red indicates up-regulation of a gene and blue indicates down-regulation of a gene. The dendrogram indicates the degree to which the expression profiles of individual replicates are similar.

Consistent with the PCA, the hierarchical clustering found high reproducibility in transcriptome changes for each of the mercurial treatments, indicating high quality data (Figure 4). Hierarchical clustering analysis of differentially expressed genes also found that the two mercurials had different effects on the *C. elegans* transcriptome (Figure 4). Gene expression profiles for sub- and low-toxicity treated nematodes were similar for the individual mercurials. The effect of sub- and low-toxicity HgCl_2_ treatments on gene transcription was nearly opposite to the effect of sub- and low-toxicity MeHgCl treatments. Genes up-regulated by HgCl_2_ sub- and low-toxicity treatments were down-regulated by MeHgCl treatments, and genes down-regulated by sub- and low-toxicity HgCl_2_ treatments were up-regulated by sub- and low-toxicity MeHgCl treatments (Figure 4). The gene expression profiles for the high-toxicity exposures for HgCl_2_ and MeHgCl were both dissimilar from the other treatments. There were, however, a small number of common differentially expressed genes at the highest HgCl_2_ and MeHgCl concentrations. These may represent a general stress response that could be induced as the nematodes begin to succumb to mercurial toxicity. The PCA and hierarchical clustering results suggest that changes in transcription are dictated largely by the type of mercurial.

Microarray results were further analyzed to identify biological processes affected by the mercurials. Differentially expressed genes were examined for enrichment in Gene Ontologies. Table 4 lists the significantly enriched GO biological processes for each mercurial treatment. For genes affected by high-toxicity HgCl_2_ and MeHgCl exposures, there was an enrichment of genes involved in lipid glycosylation and body morphogenesis. Aside from this difference, there was very little overlap in enriched GO processes between mercurials. There was not a significant enrichment of genes down-regulated in low-toxicity HgCl_2_ exposures. The lack of overlap in enriched GO terms further indicated that HgCl_2_ and MeHgCl had different effects on gene expression and affect unique biological processes.

**Table 4:** Enriched GO biological processes for differentially expressed genes

**Mercurial**	**Toxicity**	**Change in expression**	**Biological process (p-value)**
MeHgCl	Sub-toxic	up-regulated	Metabolic process (0.00097)
	Low-toxic	up-regulated	Lipid glycosylation (4.6E-05)
			Response to heat (0.00084)
			Carbohydrate metabolic process (0.0084)
			Oxidation reduction (0.04)
		down-regulated	Regulation of transcription (0.032)
	High-toxic	up-regulated	Post-translational protein modification (3.4E-67)
			Vitelline membrane formation (8.5E-07)
			Lipid glycosylation (1.1E-05)
			Response to heat (0.0022)
			Enterobactin biosynthetic process (0.044)
		down-regulated	Cell adhesion (9.5E-06)
			Body morphogenesis (1.8E-05)
			Cell-matrix adhesion (4.7E-05)
			Chitin catabolic process (0.00021)
			Regulation of transcription (0.0012)
			Cell wall macromolecule catabolic process (0.025)
			Defense response (0.0035)
			Tail morphogenesis (0.0044)
			Proteolysis (0.0046)
			Cilium morphogenesis (0.012)
			Neuron recognition (0.021)
			Lipid transport (0.021)
			Ubiquitin-dependent catabolic process (0.032)
			Response to oxidative stress (0.035)
			Protein-DNA complex assembly (0.042)
			Regulation of cell migration (0.046)
HgCl_2_	Low-toxic	up-regulated	Proteolysis (7.4E-06)
	High-toxic	up-regulated	Lipid glycosylation (2.5E-14)
			Transmembrane transport (1.1E-06)
			Carbohydrate metabolic process (2.1E-06)
			Extracellular matrix organization (1.3E-05)
			Oxidation reduction (0.0084)
		down-regulated	Body morphogenesis (6.8E-10)
			Locomotion (0.018)
			Morphogenesis of an epithelium (0.024)
			Regulation of multicellular organism growth (0.036)

### Co-expressed genes

**Figure 5:**
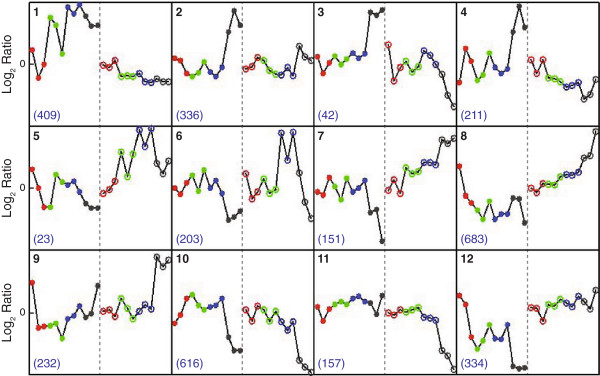
**EPIG clustering of co-expressed genes.** The average log_2_ fold-change of genes in the pattern for each experimental replicate and treatment is represented by the individual data points in each box. Red, green, blue, and black data points are untreated, sub-, low-, and high-toxicity exposures, respectively. Data points to the left of the dotted line (closed circles) are HgCl_2_-responsive genes and those to the right (open circles) are MeHgCl-responsive. The value in the upper left indicates the identification number of the EPIG pattern. The value in the lower left hand indicates the number of genes in the specific pattern. The identity of the genes in each pattern can be found in Additional file 3: Table S5.

The EPIG analysis tool identified gene expression patterns that differed between the two mercurials and the treatment concentrations and then categorized genes with similar patterns of transcription [29]. It has been proposed that genes with similar expression patterns across different treatment conditions may be co-regulated or involved in related biological processes. Using data for all probes and mercurial treatment conditions, 12 unique expression patterns were generated (Figure 5). The number of genes assigned to each pattern varied from 23 genes (pattern 5) to 683 (pattern 8). In each pattern, HgCl_2_ and MeHgCl exposure had different effects on gene expression, which further demonstrated the extent to which HgCl_2_ and MeHgCl had different effects on transcription. A list of the genes in the different EPIG patterns can be found in Additional file 3: Table S5.

Bioinformatics analyses of genes in different EPIG patterns elucidated the biological processes affected by HgCl_2_ and MeHgCl exposure. The efficacy of EPIG in grouping genes with related biological processes was evinced by the greater number of significantly enriched GO categories relative to analysis of the total DEGs. Among the 12 EPIG patterns, there were 104 significantly enriched GO categories. By comparison, analysis of DEGs by treatment and differential expression yielded 38 significantly enriched GO categories. Examining the pattern-specific GO categories defined how *C. elegans* responded to mercurials, and how these responses differed. The five most significantly enriched GO biological processes for each EPIG pattern are listed in Table 5. All significantly enriched biological process and molecular function GO categories are listed in Additional file 4: Table S6.

**Table 5:** Significantly affected GO biological process in each EPIG pattern

**EPIG pattern**	**Biological process (p value)**
1	Oxidative phosphorylation (0.00013)
	Ion transport (0.00020)
	Response to drug (0.00024)
	Monovalent inorganic cation transport (0.00031)
	Metal ion transport (0.00032)
2	Lipid glycosylation (0.0011)
	Monovalent inorganic cation transport (0.0043)
	Metal ion transport (0.0045)
	Transport (0.026)
3	Lipid storage (0.033)
4	Defecation (3.4E-05)
	Oxygen transport (0.00029)
	Regulation of pharyngeal pumping (0.00037)
	Transmembrane transport (0.0084)
	Neurotransmitter transport (0.021)
5	Cytokinesis (1.5E-06)
	Embryonic development ending in birth or egg hatching (0.045)
6	Meiosis (0.018)
7	Embryonic development ending in birth or egg hatching (6.1E-08)
	Mitotic spindle organization (1.2E-05)
	Reproduction (1.8E-05)
	Cytokinesis (0.00027)
	Germ cell development (0.0051)
8	Embryonic development ending in birth or egg hatching (1.4E-28)
	Nematode larval development (7.1E-14)
	Genitalia development (1.0E-12)
	Receptor-mediated endocytosis (2.7E-11)
	Protein catabolic process (1.3E-8)
9	tRNA aminoacylation for protein translation (1.1E-06)
	Lipid glycosylation (0.0020)
	Positive regulation of growth rate (0.033)
10	Lipid metabolic process (0.00073)
	Positive regulation of programmed cell death (0.0019)
	Lipid transport (0.0028)
	Body morphogenesis (0.0047)
	Cell adhesion (0.0078)
11	Oviposition (0.014)
	Transmembrane transport (0.020)
12	Embryonic development ending in birth or egg hatching (4.2E-16)
	Cell division (9.2E-10)
	Morphogenesis of an epithelium (1.3E-6)
	Mitotic spindle organization (4.1E-6)
	Embryonic pattern specification (2.3E-5)

The GO category *embryonic development associated with birth or egg hatching* was significantly enriched in EPIG patterns 5, 7, 8, and 12. However, that ontology comprised 3,555 genes, which were involved in a variety of functions. Patterns 1 and 2 included genes that were up-regulated in response to HgCl_2_, but were down-regulated or not affected by MeHgCl. In both patterns, there was an enrichment of genes in the *metal transport* GO category, which included a number of potassium channels (*ccb-2*, *egl-23*, *irk-2*, *kcnl-2*, *shl-1*, *slo-2*, *twk-29*, *twk-33*, *twk-35*, *twk-43*, *twk-46*, *unc-8*, C53A5.5). Potassium channels have been reported to be inhibited by HgCl_2_ and unaffected by MeHgCl exposure [30,31]. It is possible that HgCl_2_, but not MeHgCl, inhibited potassium channel activity in *C. elegans*, and that the nematode responded by increased transcription of the affected proteins. However, further investigation is required to determine if this is the case.

Pattern 8 comprised 683 genes that were down-regulated in response to HgCl_2_ and up-regulated in response to MeHgCl. There was a significant enrichment of genes in the *protein catabolic process*, including components of the proteasome (*pas-1*, *pas-2*, *pas-3*, *pas-4*, *pas-5*, *pas-6*, *pas-7*, *pbs-2*, *pbs-4*, *pbs-6*, *pbs-7rpt-1*, *rpt-2*, *rpt-3*, *rpt-5*, *rpt-6*), ubiquitin ligases (*skr-2* and *skr-18*), and ubiquitin-specific proteases (*usp-14*, *usp-48*, *usp-5*). This suggested that nematodes responded to an increase in the level of methylmercury-damaged proteins by up-regulating the ubiquitin-proteasome system.

Pattern 9 contained 232 genes whose levels of expression increased at high-toxicity MeHgCl exposures, but were largely unaffected by sub- and low-toxicity MeHgCl and all HgCl_2_ exposures. The most significantly enriched GO was *tRNA aminoacylation for protein translation*, which included the tRNA synthetases for asparagine, aspartic acid, glycine, methionine, serine, tyrosine and valine. MeHgCl inhibits protein synthesis, which has been attributed to the ability of MeHgCl to disrupt aminoacyl-tRNA synthetase activity [32]. The data in this report suggested that nematodes increased transcription of aminoacyl-tRNA synthetases to compensate for the inhibition of these enzymes by MeHgCl.

### Functional analysis of mercury-responsive *C. elegans* genes

Exposure to HgCl_2_ and MeHgCl resulted in the up-regulation of hundreds of *C. elegans* genes. We hypothesized that up-regulated genes were likely to be important in protecting *C. elegans* against mercurial toxicity. To investigate this hypothesis, RNAi was used to assess the effects of knocking down gene expression on *C. elegans* growth in the presence of HgCl_2_ or MeHgCl. Genes whose level of expression increased > 2-fold under all HgCl_2_ exposure conditions and the sub- and low-toxicity MeHgCl exposures were selected. In addition, genes whose level of expression increased > 5-fold at the high-toxicity MeHgCl exposure were selected. Using these selection criteria, 599 genes were tested, which included 258, 276, and 65 genes that were up-regulated by HgCl_2,_ MeHgCl, and both mercurials, respectively.

Gene-mercurial interactions were tested for both mercurials for all genes. An interaction was identified when gene knockdown and mercurial exposure resulted in growth that was significantly different from the predicted additive effects of the independent mercurial exposure and knockdown in gene expression.

In the initial screen, significant gene-mercurial interactions to at least one mercurial for 155 genes were observed. The effect on growth of knocking down these genes concomitant with mercurial exposure was then quantitatively assessed. For each gene-mercurial combination, interaction parameters and p-values were calculated. A positive interaction parameter indicated that nematodes exposed to mercurial and dsRNA had greater than predicted growth (i.e. knocking down the gene increased the nematodes’ resistance to the mercurial). A negative interaction indicated that nematodes exposed to both mercurial and dsRNA had less than predicted growth (i.e. knocking down the gene decreased the nematodes’ resistance to that mercurial).

Eighteen genes showed significant gene-mercurial interactions (Table 6). Genes that had positive gene-mercurial interactions included those encoding choline kinase (*ckb-2*), an F-box A protein (*fbxa-136*), an ATP-binding cassette transporter (*wht-3*), and a C-type lectin (*clec-163*). Genes that had negative gene-mercurial interactions included genes encoding: γ-glutamylcysteine synthetase (*gcs-1*), a member of the tetraspanin integral membrane protein family (*tsp-5*), a poly-ADP-ribose metabolism enzyme (*pme-4*), an F-box A protein (*fbxa-116*), and a long-chain fatty acid elongation enzyme (*elo-6*). The remaining genes have limited information related to their function. Of the 20 significant interactions, two genes showed significant gene-mercurial interactions with both mercurials: *gcs-1* and F14F9.4, which encodes a hypothetical protein. The remaining gene-mercurial interactions were mercurial-specific. The results of all *C. elegans* gene-mercurial interactions are listed in Additional file 5: Table S7.

**Table 6:** ***C. elegans *** **gene-mercurial interactions**

**Mercurial**	**Sequence name**	**Gene name**	**Interaction p-value**	**Interaction parameter**
HgCl_2_	F14F9.4		0.00072	161
	F59D6.2		0.00036	129
	B0285.9	*ckb-2*	1.20E-06	122
	C54D10.8		0.0022	122
	C18D4.2	*fbxa-136*	0.0036	107
	F19C7.5		3.00E-06	95
	C16C10.12	*wht-3*	0.00022	94
	Y39A1B.1	*clec-163*	0.0017	78
	T09F5.10		0.0061	65
	F37B12.2	*gcs-1*	2.40E-05	-228
MeHgCl	F14F9.4		0.0068	121
	K01D12.1		0.0024	54
	F37B12.2	*gcs-1*	2.9E-11	-354
	Y45F10B.1	*tsp-5*	0.0016	-153
	H23L24.5	*pme-4*	0.0069	-148
	Y113G7B.1	*fbxa-116*	0.00057	-137
	Y69E1A.8		0.0021	-104
	T05E7.4		0.00013	-103
	T22D1.2		0.00079	-101
	F41H10.8	*elo-6*	0.010	-99

### Effect of mercurials on gene expression in human cells

Results with *C. elegans* indicated that transcriptional responses varied by type of mercurial. To determine if a similar response occurred in human cells, the effects of HgCl_2_ and MeHgCl exposure on transcription in three human-derived cell lines: SK-N-SH, HepG2 and HEK293 were examined. The effects on the steady state mRNA levels of human homologs to *C. elegans* genes for which there were significant gene-mercurial interactions were determined: ABCG2 (*C. elegans* WHT-3), a transporter that exports chemotherapeutic agents from cells and is up-regulated in many cancers [33]; BACE1 (*C. elegans* ASP-7), the rate limiting enzyme for the production of β-amyloid peptide [34]; BACE2, a BACE1 homolog, though less is known about its function; choline kinases CHKA and CHKB (*C. elegans* CKB-2), generate phosphocholine [35]; ELOVL3 and ELOVL6 (*C. elegans* ELO-6), synthesize saturated and mono-unsaturated long-chain fatty acids [36]; GCLC (*C. elegans* GCS-1), glutamate-cysteine ligase catalytic subunit for the rate limiting enzyme in glutathione synthesis [37]; and PARG (*C. elegans* PME-4), catalyzes the hydrolysis of poly-(ADP-ribose) [38].

A total of 162 cell line-mercurial concentration-gene combinations were tested. Of these, 36 resulted in a significant affect on gene expression, and every gene was differentially expressed in at least one condition (Table 7). Interestingly, while the *C. elegans* homologs of these genes were all up-regulated in response to at least one mercurial, many of the human homologs were down-regulated. This was evident in the HepG2 cells, where PARG, ELOVL6, BACE1, CHKA, CHKB, and ABCG2 were down-regulated in response to MeHgCl exposure. There were also cell line specific responses to mercurial exposure. For example, BACE2 was up-regulated at the HgCl_2_ EC_20_ and EC_50_ in SK-N-SH cells, but was down-regulated at the EC_50_ in HEK293 cells.

**Table 7:** Effects of mercurial on gene expression in cells

**Genes**		**SK-N-SH**						**HepG2**						**HEK293**					
***C. elegans***	**Human**	**HgCl**_**2**_			**MeHgCl**			**HgCl**_**2**_			**MeHgCl**			**HgCl**_**2**_			**MeHgCl**		
		**NOAEL**	**EC**_**20**_	**EC**_**50**_	**NOAEL**	**EC**_**20**_	**EC**_**50**_	**NOAEL**	**EC**_**20**_	**EC**_**50**_	**NOAEL**	**EC**_**20**_	**EC**_**50**_	**NOAEL**	**EC**_**20**_	**EC**_**50**_	**NOAEL**	**EC**_**20**_	**EC**_**50**_
*wht-3*	ABCG2	Not Detected						-	-	-	-2.0	-3.0	-5.3	-	-	1.9	-	-	-
*asp-7*	BACE1	-	-	-	-	-	-	-	-	-	-	-1.9	-3.1	-	-	-	-	-	-
	BACE2	-	1.8	2.8	-	-	-	-	-	-	-	-	-	-	-	-1.7	-	-	-
*ckb-2*	CHKA	-	-	3.8	-	-	-	-	-	-1.5	-	-1.4	-2.3	-	-	1.9	-	-	-
	CHKB	-	-	2.0	1.8	2.7	2.0	-	-	-	-1.2	-	-2.0	-	-	-	-	-	-
*elo-6*	ELOVL3	-	-	9.8	-	-	-	-	-	-	-	11	10	-	-	8.3	-	-	7.0
	ELOVL6	-	-	1.8	-	-	-	-	-2.0	-	-2.2	-2.4	-2.8	-	-	-	-	-	-
*gcs-1*	GCLC	-	-	2.6	-	-	-	2.5	2.6	-	2.8	2.1	-	-	-	-	-	-	-
*pme-4*	PARG	-	-	-	-	-	-	-	-	-	-	-	-2.4	-	-	-	-	-	-

The number in each cell indicates the fold-change in expression. Cells containing ”-“ indicate no significant change in gene expression.

As was observed in *C. elegans*, HgCl_2_ and MeHgCl had different effects on transcription. For example, in HepG2 cells, both EC_20_ and EC_50_ MeHgCl treatments resulted in an ~10-fold increase in ELOVL3 levels, while HgCl_2_ exposure had no effect on ELOVL3 mRNA levels. Of the 36 conditions that resulted in a significant change in gene expression, 24 were unique to a specific cell line-mercurial combination. There were six conditions where both mercurials, at equitoxic concentrations, induced similar changes in gene expression. In SK-N-SH cells, CHKB was up-regulated by EC_50_ exposures to both mercurials, and in HEK293 cells, ELOVL3 was up-regulated by EC_50_ exposures to both mercurials. In HepG2 cells, GCLC was up-regulated by NOAEL and EC_20_ HgCl_2_ and MeHgCl treatments, while ELOVL6 and CHKA were down-regulated by EC_20_ and NOAEL treatments, respectively. There were no instances in which a gene was significantly up-regulated by one mercurial and significantly down-regulated by the other. Overall, these results were similar to that observed in *C. elegans*, where HgCl_2_ and MeHgCl exposure showed metal-specific effects on gene expression.

### Functional analysis of gene-mercurial interactions of human homologs

A subset of *C. elegans* genes up-regulated in response to mercurial exposure was found to be important in the nematode response to mercurial exposure. To determine if the human homologs of these genes also affected the mammalian response to mercurial exposure, the effect of gene knockdown on the viability of SK-N-SH, HepG2 and HEK293 cells after a 24 h exposure to estimated EC_50_ mercurial concentrations was determined. There was no detectable ABCG2 expression in SK-N-SH cells, and BACE2 was not significantly knocked down in SK-N-SH and HepG2 cells, therefore these conditions were not tested. In all other cases, siRNA treatment resulted in a significant decrease in target mRNA (Additional file 1: Figure S4).

As with the *C. elegans* RNAi experiment, genes were deemed critical to the cells’ response to mercurial exposure if there was a significant gene-mercurial interaction. A positive interaction indicated more than expected viable cells, and a negative interaction indicated fewer than expected viable cells. There were 11 significant interactions (Table 8). There were no significant interactions with either mercurial for BACE1, BACE2, or CHKB in any cell line. There were no instances in which a gene-cell line combination resulted in a significant interaction with both HgCl_2_ and MeHgCl. Ten of the significant interactions were negative, with only knockdown of ELOVL6 in HgCl_2_-treated HepG2 cells resulting in a positive interaction. This interaction resulted in a 58% increase in viable cells, which was the largest magnitude change of any gene-mercurial interaction. Knockdown of ELOVL3 resulted in negative interactions in HgCl_2_-treated SK-N-SH cells and MeHgCl-treated HEK293 cells, and had no effect in mercurial-treated HepG2 cells. Knockdown of CHKA in MeHgCl-exposed cells resulted in a negative interaction in all three cell lines. However, significant CHKA-HgCl_2_ interactions were not observed. Knockdown of ABCG2 in MeHgCl-exposed cells resulted in negative interactions in HepG2 and HEK293 cells. All other gene-mercurial interactions were cell line-specific. There was a significant GCLC-mercurial effect only in SK-N-SH cells treated with HgCl_2_ and HepG2 cells treated with MeHgCl. As the GCLC homolog, *gcs-1*, was the most critical resistance gene to both mercurials in *C. elegans*, it was expected that similar results would be observed in cell culture. As was observed in *C. elegans*, different genes are critical in the cellular response to different mercurials.

**Table 8:** Gene-mercurial interactions in human cells

**Gene**	**Mercurial**	**Interaction parameters (p-values)**		
		**SK-N-SH**	**HepG2**	**HEK293**
ABCG2	HgCl_2_	NA	-1% (0.83)	-5% (0.56)
	MeHgCl	NA	**-8% (0.038)**	**-28% (0.0068)**
BACE1	HgCl_2_	-21% (0.057)	21% (0.069)	-1% (0.92)
	MeHgCl	-11% (0.22)	-3% (0.68)	-28% (0.063)
BACE2	HgCl_2_	NA	NA	-2% (0.87)
	MeHgCl	NA	NA	-26% (0.30)
CHKA	HgCl_2_	-26% (0.088)	-3% (0.72)	-20% (0.11)
	MeHgCl	**-27% (0.037)**	**-21% (0.0028)**	**-31% (0.021)**
CHKB	HgCl_2_	-18% (0.39)	19% (0.24)	4% (0.71)
	MeHgCl	-10% (0.24)	1% (0.92)	5% (0.78)
ELOVL3	HgCl_2_	**-21% (0.0066)**	-4% (0.76)	-7% (0.62)
	MeHgCl	-5% (0.53)	5% (0.35)	**-37% (0.0023)**
ELOVL6	HgCl_2_	-25% (0.35)	**58% (<0.001)**	3% (0.91)
	MeHgCl	-28% (0.071)	3% (0.57)	20% (0.43)
GCLC	HgCl_2_	**-15% (0.031)**	0% (1.00)	-25% (0.060)
	MeHgCl	-2% (0.45)	**-22% (<0.001)**	3% (0.82)
PARG	HgCl_2_	-15% (0.15)	-4% (0.59)	-22% (0.20)
	MeHgCl	6% (0.61)	1% (0.96)	**-53% (0.0076)**

Significant (at the 0.05 alpha cutoff) positive and negative gene-mercurial interactions are highlighted in **bold**. Numbers in cells indicate the percent change in viable cells from predicted additive effect of gene knock-down and mercurial exposure. “NA” indicates a condition that was not tested.

## Discussion

In the present study, global transcriptome profiles for *C. elegans* exposed to sub-, low- and high-toxicity concentrations of HgCl_2_ and MeHgCl were compared. The use of three equitoxic levels of mercurial allowed for a more relevant comparison of the effects. After demonstrating the differences in *C. elegans* responses to HgCl_2_ and MeHgCl exposure, the role of selected genes in mercurial response was assessed in three human cell lines. In both *C. elegans* and mammalian cells, HgCl_2_ and MeHgCl exposure had unique effects on gene expression, and different genes were important in protecting the organism from mercurial toxicity.

At each equitoxic exposure, there were a greater number of DEGs in MeHgCl-treated *C. elegans* than in HgCl_2_-treated. Furthermore, at each level of toxicity, there was a higher percentage of DEGs down-regulated by MeHgCl, compared to HgCl_2_: sub-toxicity (0% HgCl_2_, 20% MeHgCl), low-toxicity (8% HgCl_2_, 41% MeHgCl), high-toxicity (22% HgCl_2_, 43% MeHgCl). High percentages (41%-76%) of DEGs were down-regulated in response to MeHgCl in studies using mouse embryo fibroblasts [39]. In contrast, microarray analysis of livers from HgCl_2_-exposed zebrafish found approximately equal numbers of up- and down-regulated genes, and analysis of Hg^0^-exposed rat lungs found more up-regulated than down-regulated genes [22,40].

One of the most striking results of the present study was the degree to which the transcriptomes of HgCl_2_ and MeHgCl differed. There were very few common DEGs between mercurial exposures. PCA indicated that more variability was attributed to the mercurial species than the level of toxicity (Figure 3). Similarly, hierarchical clustering of DEGs at sub-and low-toxicity concentrations showed almost opposite transcriptional responses for HgCl_2_ and MeHgCl (Figure 4). At high-toxicity exposures, when cellular stress is elevated, one would expect similarities in the DEGs. There were 94 up-regulated and 14 down-regulated common DEGs following exposure to high-toxicity concentrations of HgCl_2_ and MeHgCl. While the majority of these genes were poorly characterized, there were several known stress-response genes: seven UDP-glucuronosyl transferases, six glutathione *S*-transferases, and a heat shock protein. The common DEGs, however, represented less than 3.5% of the total number of DEGs. The differences in the HgCl_2_ and MeHgCl transcriptomes suggest that the two mercurials have different mechanisms of action.

HgCl_2_ and MeHgCl differed in the number of positive and negative gene-mercurial interactions. Of the 18 genes for which there was a gene-mercurial interaction, only two interacted with both HgCl_2_ and MeHgCl. Of the ten significant gene-MeHgCl interactions, eight resulted in increased susceptibility to MeHgCl. Conversely, of the ten significant gene-HgCl_2_ interactions, nine resulted in increased tolerance to HgCl_2_. The differences in direction of the gene-mercurial interactions between the two mercurials and the paucity of similar gene-mercurial interactions were further evidence that the two mercurials acted through unique mechanisms at the molecular level.

Metallothionein binding of inorganic metals is a common detoxification mechanism. Therefore, increased mRNA levels were expected following mercurial exposure. The *C. elegans* metallothionein genes, *mtl-1* and *mtl-2*, were up-regulated in response to HgCl_2_ exposure. They were however, down-regulated following MeHgCl exposure. Previous publications in a variety of species report inconsistent induction of metallothioneins in response to MeHgCl exposure [41-44]. The present study is the first to report a down-regulation of metallothionein gene expression in response to MeHgCl exposure. Although earlier studies showed that metallothioneins were incapable of binding methylmercury [45], more recent work indicated that metallothioneins could bind methylmercury [46]. In addition to binding metals, metallothioneins are also important in regulating the redox status of cells and preventing intracellular oxidative damage. Metallothioneins increase resistance to MeHgCl toxicity, possibly by protecting cellular components from mercurial-induced oxidative stress [47]. These data suggest that the increased sensitivity of *C. elegans* to MeHgCl may be a consequence of its inability to induce metallothionein expression in response to this mercurial.

In MeHgCl-exposed *C. elegans*, decreased metallothionein levels likely result in increased reliance on glutathione-mediated detoxification. In the low-toxicity MeHgCl treatment, 13 glutathione *S*-transferases were up-regulated. Conversely, no glutathione *S*-transferases were up-regulated in the low-toxicity HgCl_2_ exposure. In high-toxicity treatments, there were 19 glutathione *S*-transferases up-regulated in MeHgCl-exposed nematodes and seven in HgCl_2_-exposed nematodes. In addition, knockdown of *gcs-1* increased *C. elegans* susceptibility to both mercurials; however, the effect was greater in MeHgCl-exposed nematodes (Table 6). The human homolog of *gcs-1*, GCLC, was also critical in resistance to both mercurials in mammalian cells. Knockdown of GCLC resulted in significant negative interactions with HgCl_2_ in SK-N-SH and MeHgCl in HepG2 cells. Glutathione is important in resistance to both HgCl_2_ and MeHgCl, but MeHgCl-exposed *C. elegans* appear to be particularly dependent on glutathione-mediated resistance. Gene expression and knockdown results with both *C. elegans* and human cells suggest that glutathione may be a component of an evolutionarily conserved defense against mercurial (organic and inorganic) toxicity.

Co-exposure of PARG siRNA and MeHgCl in HEK293 cells resulted in the second largest observed gene-mercurial interaction, indicating the critical role of PARG in resistance to MeHgCl toxicity. In contrast, there were no significant PARG-HgCl_2_ interactions in any cell line. PARP (poly-ADP-ribose polymerase) catalyzes the addition of ADP-ribose to proteins, while PARG cleaves poly-ADP-ribose to ADP-ribose monomers [48]. In cases of severe stress, PARP becomes highly activated, which leads to over-production of poly-ADP-ribose and cell death [49]. This suggests that exposure to MeHgCl increases PARP activity, and that PARG is necessary to maintain poly-ADP-ribose homeostasis. Treatment with the PARP inhibitor 3, 4-dihydro-5-[4-(1-piperidinyl)-butoxy]-1(2*H*)-isoquinolinone decreased MeHgCl-induced cell death in a dose-dependent manner [50]. PME-4, the *C. elegans* PARG homolog, was up-regulated 22-fold in low-toxicity and 35-fold in high-toxicity MeHgCl exposures. In addition, *pme-4* knockdown during MeHgCl exposure resulted in the fourth greatest negative interaction, however, *pme-4* knockdown during HgCl_2_ exposure did not produce a significant interaction. PME-4 is primarily expressed in the cytoplasm of neurons, and is predicted to be critical in preventing neurodegeneration [51]. Methylmercury is a neurotoxicant, thus PME-4 may be critical in maintaining neuron viability in MeHgCl-exposed nematodes. These results suggest that disruption of poly-ADP-ribose homeostasis may be an evolutionarily conserved mechanism of MeHgCl, but not HgCl_2_, toxicity.

ELO-6 was important in resistance to MeHgCl, though it was down-regulated 2.5-fold in the high-toxicity MeHgCl exposure. ELO-6 is a long-chain fatty acid elongation enzyme that plays an essential role in growth of *C. elegans*[52]. There is evidence that suggests exposure to poly-unsaturated fatty acids mitigates MeHgCl toxicity in humans [53,54].

EPIG pattern 8 was populated by genes down-regulated by HgCl_2_ and up-regulated by low- and high-toxicity MeHgCl exposures. There was a significant enrichment of genes involved in the ubiquitin-proteasome system in this group. In yeast, increased ubiquitination resulted in increased resistance to MeHgCl toxicity [55,56]. In addition, MeHgCl-exposed mouse embryonic fibroblasts showed an enrichment of differentially expressed genes involved in the ubiquitin-proteasome system. These authors suggested that the removal of methylmercury-damaged proteins is critical for cell survival [23]. Recent work indicates that ubiquitination inhibits neuronal exit in cerebellar granule cell layers [57]. Patients suffering from Minamata disease exhibited hypoplasia of granule cell layer and degeneration of cerebellar granule cells, which may be due in part to MeHgCl inhibition of granule cell migration [58,59]. It is reasonable to hypothesize that increased ubiquitination, while protective against MeHgCl in some cell types, may contribute to the neuropathology of methylmercury exposure by inhibiting granule cell migration.

In the present study, considerable differences were observed on how exposure to different mercurials affects gene expression and the genes that mediate the organism’s response to mercury. These differences were observed in both *C. elegans* and human cell lines, demonstrating the conserved nature of this phenomenon. While there are many instances in which a specific gene was differentially expressed in response to only one mercurial there were no instances in which a gene was up-regulated by one mercurial and down-regulated by the other. These results suggest that, despite similarities in the known responses of the two mercurials, the two mercurials function differently at the molecular level. These data indicate that contrary to previous models that hypothesize the conversion of organic mercury to the inorganic species, organic and inorganic mercurials act by independent or unique molecular mechanisms.

## Conclusion

Mercury is an environmental human toxicant that exists in multiple chemical forms. Despite years of research, only a fragmented understanding of the molecular mechanisms of mercurial toxicity exists. Furthermore, it is not known whether different mercurial species act similarly or dissimilarly at the molecular level. We performed microarray analysis of *C. elegans* exposed to three equitoxic concentrations of mercuric chloride (HgCl_2_) and methylmercury chloride (MeHgCl). Bioinformatics analysis indicated that the transcriptional effects of the two mercurials were vastly different. Next, we examined the effects of knocking down the expression of up-regulated genes on *C. elegans* growth. Of the ~600 genes tested, only two significantly affected growth in response to both mercurials. We examined the evolutionarily conserved nature of the mercurial response in three human-derived cell lines. Similar to what was observed in *C. elegans*, there was very little overlap in the transcriptional responses between HgCl_2_ and MeHgCl. There was also very little overlap between the mercurials in the genes involved in resistance and susceptibility. These data indicate that contrary to previous models that hypothesize the conversion of organic mercury to the inorganic species, which is the active form of the metal, organic and inorganic mercurials function by independent or unique molecular mechanisms.

## Methods

### Maintenance of *C. elegans*

Wild-type N2 Bristol and NL2099 (*rrf-3(pk1426)* II) strains were obtained from the *C. elegans* Genetic Center. *C. elegans* were maintained at 20°C on K-agar plates with *Escherichia coli* OP50 as food [60].

### *C. elegans* treatment and RNA isolation

Forty L4 stage N2 nematodes were placed on K-agar plates and allowed to grow for 4 d at 20°C. Nematodes were then transferred into liquid S-medium containing *E. coli* OP50, and incubated at 20°C with constant agitation for 4 d [61]. Fifty-milliliter aliquots of mixed-stage *C. elegans* were then used for untreated control samples, or treated with sub- (2.0 μM HgCl_2_, 0.75 μM MeHgCl), low- (7.5 μM HgCl_2_, 2.0 μM MeHgCl) or high- (20 μM HgCl_2_, 7.5 μM MeHgCl) toxicity mercurial concentrations for 24 h. Nematodes were then collected by centrifugation and rinsed three times with 0.1 M NaCl. Nematodes were isolated by sucrose floatation, frozen in liquid nitrogen, and then stored at -80°C until used, as previously described [62].

To prepare total RNA, nematodes were ground to a fine powder in a liquid nitrogen-cooled mortar and pestle, and RNA was isolated using the RNeasy Midi Kit, according to manufacturer’s instructions (Qiagen, Valencia, CA). Independently treated and control *C. elegans* cultures were used to generate three biological replicates for each treatment condition. The quality of the RNA was assessed using an Agilent 2100 Bioanalyzer (Palo Alto, CA).

### Microarray experiment and data analysis

RNA was submitted to the NIEHS Microarray Group for labeling, probe hybridization and microarray scanning. Agilent *C. elegans* Gene Expression Microarrays, Ver. 1, which contain 21,000 probes encompassing all *C. elegans* open reading frames, were used in a single channel (1-color) design. Data were obtained using Agilent Feature Extraction Software (Ver. 9.5) with the 1-color default parameters. This software performed error modeling, and adjusting for additive and multiplicative noise. Rosetta Resolver® was used to identify differentially expressed genes using an error-weighted, 1-way ANOVA with a Bonferroni correction. A 2-fold change in expression, relative to untreated controls, and a p-value < 0.01 was required for a gene to qualify as significantly, differentially expressed.

The EPIG pattern analysis tool was used to compare the transcription profiles of genes across different treatments [29]. In EPIG, the expression of a gene in each replicate was compared to the average expression of that gene in all untreated replicates. Genes with similar expression patterns were grouped together using the following parameters: correlation value (0.64), signal-to-noise ratio (3.0), and magnitude of change (0.5). Expression patterns were based on the six most highly correlated genes for each pattern.

Hierarchical clustering and principal components analysis (PCA) were performed using an agglomerative clustering method with Euclidean dissimilarity and a correlation dispersion matrix and normalized eigenvector scaling, respectively. Hierarchical clustering and PCA were performed using Partek Genomic Suites Ver. 6.5 software (Partek Incorporated, St. Louis, MO). Gene Ontology (GO) analysis was performed using Gene Ontology Enrichment Analysis Software Toolkit (GOEAST) [63]. The listed GO terms included four or more differentially expressed genes and p-values < 0.05. P-values were the result of Fisher’s Exact Test.

### Assessing knockdown of *C. elegans* genes on growth during mercurial exposure

The effects of gene knockdown on the sensitivity of *C. elegans* to mercurials were assessed using RNAi. RNAi of selected genes was performed using the Open Biosystems (Huntsville, AL) or MRC Gene Service (University of Cambridge, UK) *C. elegans* RNAi bacterial feeding libraries [64,65]. These studies were performed using the RNAi hyper-sensitive *rrf-3* strain to increase the responsiveness of the assay [66]. EC_20_s of *rrf-3* nematodes were 10.1 μM for HgCl_2_ and 3.0 μM for MeHgCl, and were used in the RNAi studies (Additional file 1: Figure S1).

A two-generation approach was used to ensure gene knockdown throughout all *C. elegans* developmental stages. First, dsRNA-expressing bacterial cultures were grown overnight at 37°C with constant agitation. Isopropyl β-D-1-thiogalactopyranoside was added to a final concentration of 2 mM, and the incubation continued for 1 h. Bacteria were then collected and resuspended in complete K-medium [67]. Bacteria were added to appropriate wells in a 96-well plate, then nine L4 nematodes were added to each well, and incubated at 20°C for 48 h. Following this incubation, 50 L1 larvae were transferred from each well to new 96-well plates, containing fresh dsRNA-expressing bacteria and HgCl_2_ or MeHgCl. Nematodes were exposed to mercurial alone, gene-specific dsRNA alone, or mercurial and gene-specific dsRNA.

The effects of dsRNA and/or mercurial on *C. elegans* growth were assessed following a 48 h incubation. The initial assessment of gene-mercurial interactions was performed by visual observation. Any gene whose knockdown appeared to affect *C. elegans* growth, and thus a potential gene-mercurial interaction, was selected for additional analysis. All of the selected clones were sequenced to verify their identity. Of the 155 clones identified in the initial assessment, six were a different gene than described.

In the second phase of the screen, nematodes were fed dsRNA-expressing bacteria as described above. Growth was then measured using the *C. elegans* growth assay, as previously described [67]. A 2-way ANOVA was used to test for significant gene-mercury interactions using 500–800 nematodes per treatment condition. The criterion for a statistically significant interaction was p < 0.01.

### Maintenance of mammalian cell lines

Human neuroblastoma (SK-N-SH; ATCC No. HTB-11), hepatocellular carcinoma (HepG2; ATCC No. HB-8065) and embryonic kidney (HEK293; ATCC No. CRL-1573) cells were cultured in Minimum Essential Medium supplemented with 10% fetal bovine serum and 2 mM L-glutamine. Cells were grown in a humidified incubator at 37°C under 5% CO_2_ atmosphere. These cell lines represent the primary target organs of mercurial toxicity: brain for MeHgCl, kidney for HgCl_2_ and liver, which is a primary site for mercurial metabolism.

### Mercurial cytotoxicity

The toxicity of HgCl_2_ and MeHgCl to mammalian cells was determined using the Neutral Red cell viability assay, as previously described [68]. To determine the appropriate mercurial concentrations for gene expression and gene-mercurial interaction experiments, 24 h no observed adverse effect levels (NOAELs), 20% effects concentrations (EC_20_s) and 50% effects concentration (EC_50_s) for cell viability were determined for untransfected cells and those transfected with non-homologous siRNA, respectively. EC_20_s and EC_50_s were estimated from the slopes of the dose response curves (Additional file 1: Figure S2). NOAELs were defined as the highest mercurial concentration that did not result in a significant decrease in cell viability.

### Effects of mercurials on gene expression

Quantitative reverse transcription-real time-PCR (qRT-PCR) was used to measure the effects of mercurials on the steady-state mRNA levels of the following human genes: ABCG2 (ATP-binding cassette, sub-family G (WHITE), member 2), BACE1 (β-site APP cleaving enzyme 1), BACE2 (β-site APP cleaving enzyme 2), CHKA (choline kinase α), CHKB (choline kinase β), ELOVL3 (elongation of very long chain fatty acids-like 3), ELOVL6 (elongation of very long chain fatty acid-like 6), GCLC (glutamate-cysteine ligase, catalytic subunit), and PARG (poly-ADP-ribose glycohydrolase). To determine the effects of mercurials on gene expression in human cells, approximately 10^5^ cells were incubated in 6-well plates for ~24 h after which mercurials at NOAEL, EC_20_, or EC_50_ concentrations were added (Additional file 6: Table S1). After 24 h incubation, total RNA was isolated, quantified, and stored at -80°C, as described above. cDNAs were prepared and qRT-PCR performed as previously described [69]. Fold changes in mRNA levels were calculated using the ΔΔCT method using β-actin as reference mRNA [70].

The effects of mercurial exposure on the expression of *C. elegans* metallothionein genes, *mtl-1* and *mtl-2*, were also determined. qRT-PCR of *mtl-1* and *mtl-2* was performed using RNA isolated for the microarray experiments. Myosin light chain-2 mRNA (*mlc-2*) was used as reference. Results are presented as mean ± standard error (n = 3 or 4). Data were analyzed using a 1-way ANOVA with a Dunnett’s post-hoc test, with the criterion for statistical significance set at p < 0.05. Primers were designed using the open source Primer3 program and were purchased from Integrated DNA Technologies (Coralville, IA) (Additional file 6: Table S2) [71].

### Assessing the effect of gene knockdown on cell viability during mercurial exposure

Approximately 10^4^ cells in 48-well plates were transfected in medium containing Opti-MEM (20% final concentration), lipofectamine RNAiMAX (0.2% final concentration, Invitrogen) and 25 nM of the appropriate siRNA or non-homologous siRNA (Additional file 6: Table S3). Following transfection and recovery (~24 h), mercurials were added to the medium. The concentrations used for SK-N-SH cells were 21 μM for HgCl_2_ and 5 μM for MeHgCl; for HepG2 cells, 48 μM for HgCl_2_ and 30 μM for MeHgCl; and for HEK293 cells, 17 μM for HgCl_2_ and 6.8 μM for MeHgCl. Following 24 h incubation, cell viability was determined as described above. There were 3 to 5 experimental replicates for each condition.

Significance of gene-mercurial interactions was tested using a 3-way, mixed-effects ANOVA followed by a Bonferroni post-hoc test. In the ANOVA, siRNA and mercurial exposure were treated as fixed effects, and experimental day was treated as a random effect. The predicted cell survival of siRNA and mercurial co-exposure with no interaction effect was computed from an ANOVA model. The interaction parameter for each gene-mercurial condition was determined by subtracting the predicted cell survival from the experimental cell survival of the siRNA-mercurial co-exposure. This value was divided by the predicted cell survival and reported as percent change from the “no interaction” value.

## Competing interests

The authors declare that there are no financial competing interests.

## Authors’ contributions

MKM carried out the mercurial toxicity, microarray, RNAi data analysis studies and drafted the manuscript. LAH performed the PCA analysis. JWC performed the EPIG analysis. MVS assisted with the statistical analysis of the *C. elegans* mercurial toxicity data. JHF conceived and designed the experiments, and prepared the final version of the manuscript. All authors read and approved the final manuscript.

## Supplementary Material

Additional file 1: Figure S1 Effect of mercurials on *rrf-3 C. elegans* growth. Nematode size is expressed as the log of the absorbance (Extinction; EXT) of individual nematodes at the end of a 48 h exposure to HgCl_2_ (*closed circles*) or MeHgCl (*closed squares*) minus the mean absorbance of all nematodes at the beginning of exposure. **Figure S2.** Dose response curves for human cell lines. Human neuroblastoma (SK-N-SH), hepatocellular carcinoma (HepG2), and embryonic kidney (HEK293) cells were exposed to the indicated concentrations of HgCl_2_ (*closed circles*) or MeHgCl (*closed squares*) for 24 h. Cell viability was determined by Neutral Red assay. **Figure S3.** Effect of mercurial exposure on *C. elegans* population distribution. The length (TOF) of nematodes was measured to determine size. Normed counts at each size represent the fraction of the total population. The ***red line*** indicates the population distribution of mercurial-treated nematodes and the ***black line*** indicates the population distribution of untreated nematodes. *C. elegans* population distributions were determined using a COPAS Biosort as previously described as previously described. **Figure S4.** Effectiveness of siRNA in cells. SK-N-SH (*open bar*), HepG2 (*gray bar*) and HEK293 (*black bar*) cells were transfected with gene-specific siRNA or non-homologous siRNA and incubated for 24 h. Relative mRNA levels were measured using qRT-PCR. mRNA levels in cells treated with gene-specific siRNA were compared to mRNA levels in control cells to determine percent of control. Results display the mean percent of control ± SEM. Click here for file

Additional file 2: Table S4 All Differentially Expressed Genes following mercurial exposures. Click here for file

Additional file 3: Table S5 A list of the genes contained in the different EPIG patterns. Click here for file

Additional file 4: Table S6 All significantly enriched biological process and molecular function GO categories in each of the EPIG patterns. Click here for file

Additional file 5: Table S7 All *C. elegans* gene-mercurial interactions. Click here for file

Additional file 6: Table S1 NOAEL, EC20 and EC50 for Mercurials in Human Cells. **Table S2.** Nucleotide sequences of primers used for qRT-PCR. **Table S3.** Sequences of siRNA used in mammalian cell culture. Click here for file
