# Supplementary figures and images for: Comparative toxicogenomic responses of mercuric and methyl-mercury
Source: BMC Genomics. 2013 Oct 11;14:698. doi: 10.1186/1471-2164-14-698 (PMC3870996; doi:10.1186/1471-2164-14-698)

# Supplemental Figure 1

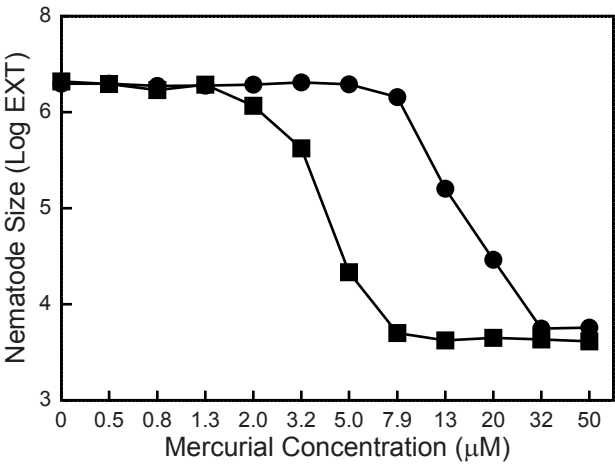

# Supplemental Figure 2

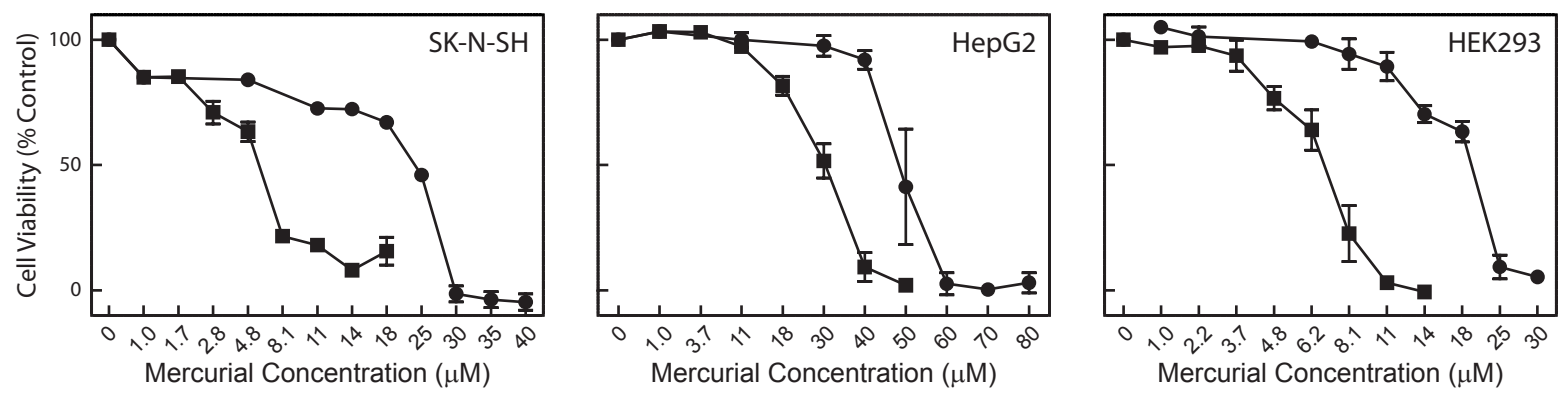

# Supplemental Figure 3

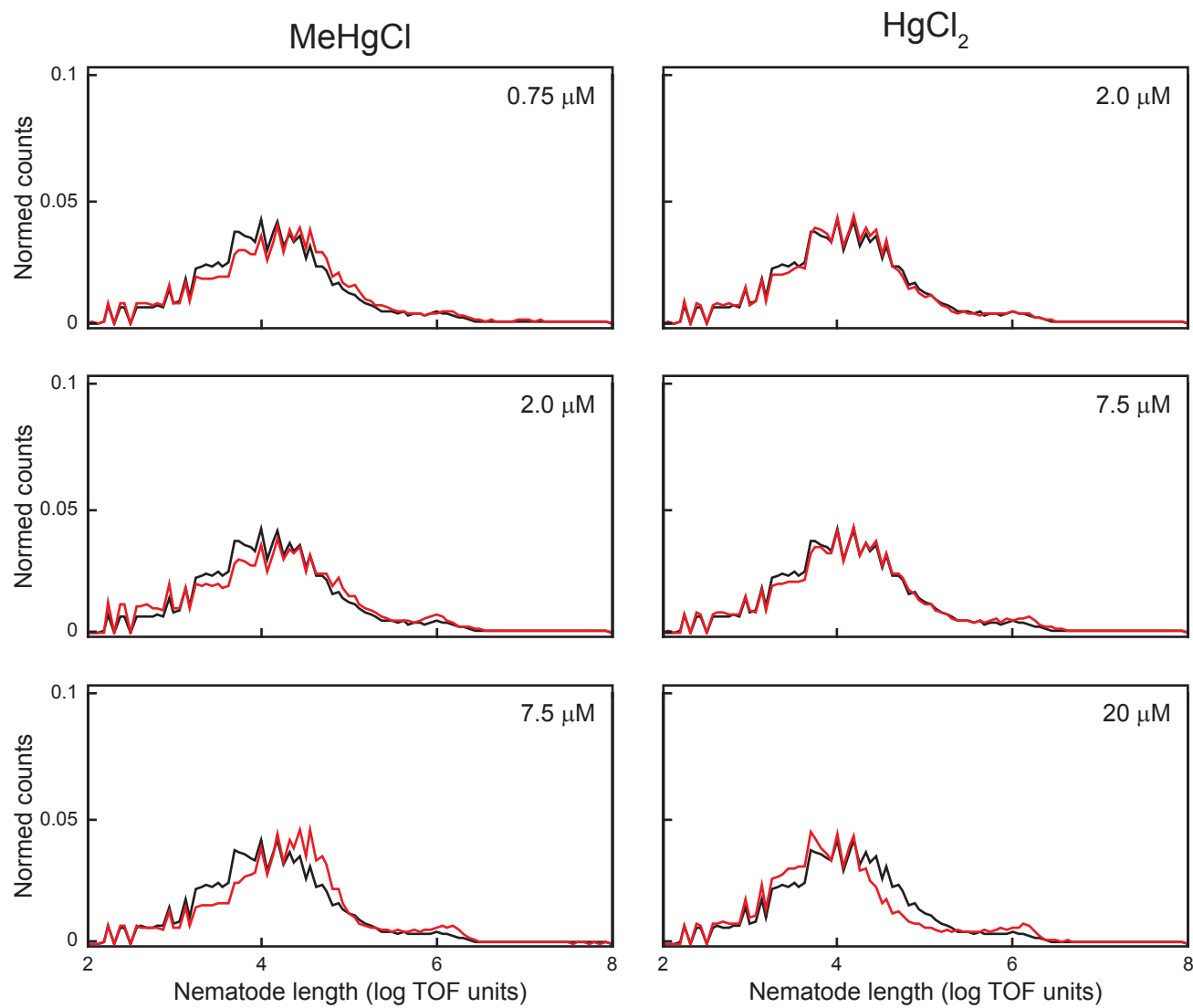

# Supplemental Figure 4

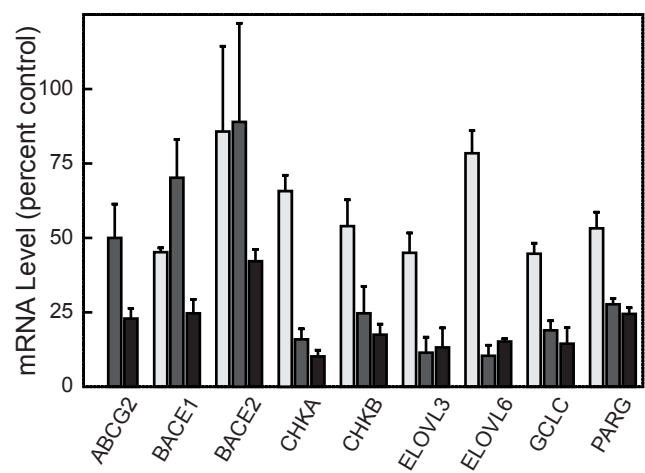

Supplement: Additional file 1: Figure S1 — Effect of mercurials on rrf-3 C. elegans growth. Nematode size is expressed as the log of the absorbance (Extinction; EXT) of individual nematodes at the end of a 48 h exposure to HgCl2 (closed circles) or MeHgCl (closed squares) minus the mean absorbance of all nematodes at the beginning of exposure. Figure S2. Dose response curves for human cell lines. Human neuroblastoma (SK-N-SH), hepatocellular carcinoma (HepG2), and embryonic kidney (HEK293) cells were exposed to the indicated concentrations of HgCl2 (closed circles) or MeHgCl (closed squares) for 24 h. Cell viability was determined by Neutral Red assay. Figure S3. Effect of mercurial exposure on C. elegans population distribution. The length (TOF) of nematodes was measured to determine size. Normed counts at each size represent the fraction of the total population. The red line indicates the population distribution of mercurial-treated nematodes and the black line indicates the population distribution of untreated nematodes. C. elegans population distributions were determined using a COPAS Biosort as previously described as previously described. Figure S4. Effectiveness of siRNA in cells. SK-N-SH (open bar), HepG2 (gray bar) and HEK293 (black bar) cells were transfected with gene-specific siRNA or non-homologous siRNA and incubated for 24 h. Relative mRNA levels were measured using qRT-PCR. mRNA levels in cells treated with gene-specific siRNA were compared to mRNA levels in control cells to determine percent of control. Results display the mean percent of control ± SEM. [file 1471-2164-14-698-S1.pdf]
