# Supplementary material for: Comparative toxicogenomic responses of mercuric and methyl-mercury
Source: BMC Genomics. 2013 Oct 11;14:698. doi: 10.1186/1471-2164-14-698 (PMC3870996; doi:10.1186/1471-2164-14-698)
Supplement: Additional file 4: Table S6 — All significantly enriched biological process and molecular function GO categories in each of the EPIG patterns. [file 1471-2164-14-698-S4.pdf]

EPIG Pattern	GO Biological Processes		GO Molecular Function	
	**Term**	**p-value**	**Term**	**p-value**
1	oxidative phosphorylation	0.00013	ion channel activity	0.00029
	ion transport	0.0002	hydrogen ion transmembrane transporter activity	0.0003
	response to drug	0.00024	acetylcholine receptor activity	0.0031
	monovalent inorganic cation transport	0.00031	NAD or NADH binding	0.0054
	metal ion transport	0.00032	extracellular ligand-gated ion channel activity	0.0078
	transmembrane transport	0.00056	oxygen binding	0.0088
	oxygen transport	0.0043	heme binding	0.015
	response to heat	0.021	calcium ion binding	0.025
	oxidation reduction	0.045	oxidoreductase activity, acting on NADH or NADPH	0.025
2	lipid glycosylation	0.0011	ion channel activity	0.001
	monovalent inorganic cation transport	0.0043	carbohydrate binding	0.0075
	metal ion transport	0.0045	ATP-dependent helicase activity	0.043
	transport	0.026	iron ion binding	0.049
			cysteine-type peptidase activity	0.049
3	lipid storage	0.033		
4	defecation	3.40E-05	oxygen binding	0.00068
	oxygen transport	0.00029	calcium ion binding	0.00069
	regulation of pharyngeal pumping	0.00037	motor activity	0.0042
	transmembrane transport	0.0084	iron ion binding	0.029
	neurotransmitter transport	0.021		
	transmission of nerve impulse	0.04		
5	defecation	3.40E-05	oxygen binding	0.00068
	oxygen transport	0.00029	calcium ion binding	0.00069
	regulation of pharyngeal pumping	0.00037	motor activity	0.0042
	transmembrane transport	0.0084	iron ion binding	0.029
	neurotransmitter transport	0.021		
	transmission of nerve impulse	0.04		
6	meiosis	0.018	nucleic acid binding	0.00015
			zinc ion binding	0.0089
			ATP binding	0.032
7	embryonic development ending in birth or egg hatching	6.14E-08	GTPase activator activity	0.005
	mitotic spindle organization	1.19E-05	cofactor binding	0.048
	reproduction	1.76E-05		
	cytokinesis	0.00027		
	germ cell development	0.0051		
	negative regulation of vulval development	0.0062		
	growth	0.0068		
	receptor-mediated endocytosis	0.012		
	nematode larval development	0.022		
	positive regulation of multicellular organism growth	0.026		
	body morphogenesis	0.029		
	hermaphrodite genitalia development	0.037		
	oogenesis	0.042		
	meiotic chromosome segregation	0.049		
8	embryonic development ending in birth or egg hatching	1.37E-28	threonine-type endopeptidase activity	1.58E-13
	nematode larval development	7.10E-14	ATP binding	1.51E-10
	genitalia development	1.02E-12	ATP-dependent helicase activity	1.23E-06
	receptor-mediated endocytosis	2.67E-11	unfolded protein binding	4.34E-06
	protein catabolic process	1.30E-08	pyrophosphatase activity	1.79E-05
	reproduction	2.17E-08	aminoacyl-tRNA ligase activity	0.0001
	ubiquitin-dependent protein catabolic process	2.72E-08	ubiquitin thiolesterase activity	0.00026
	locomotion	4.38E-07	RNA binding	0.0013
	determination of adult lifespan	7.60E-07	transcription factor binding	0.0087
	growth	7.81E-07	nuclease activity	0.026
	morphogenesis of an epithelium	1.02E-06	magnesium ion binding	0.039
	negative regulation of cell proliferation	4.61E-05		
	cell division	6.63E-05		
	protein folding	9.43E-05		
	tRNA aminoacylation for protein translation	0.00014		
	microtubule polymerization or depolymerization	0.0002		
	positive regulation of growth rate	0.0002		
	proteolysis involved in cellular protein catabolic process	0.00031		
	chromosome segregation	0.00042		
	rRNA processing	0.002		
	RNA processing	0.0022		
	pronuclear migration	0.0022		
	RNA interference	0.0023		
	protein depolymerization	0.0024		
	establishment of mitotic spindle orientation	0.0025		
	development of primary male sexual characteristics	0.0037		
	negative regulation of multicellular organism growth	0.0055		
	gastrulation with mouth forming first	0.0082		
	spermatogenesis	0.0095		
	regulation of embryonic development	0.011		
	response to DNA damage stimulus	0.012		
	meiosis	0.014		
	embryonic morphogenesis	0.016		
	body morphogenesis	0.026		
	GPI anchor metabolic process	0.029		
	microtubule-based movement	0.029		
	molting cycle, collagen and cuticulin-based cuticle	0.033		
	protein transport	0.037		
	germ cell development	0.041		
9	tRNA aminoacylation for protein translation	1.14E-06	aminoacyl-tRNA ligase activity	2.56E-06
	lipid glycosylation	0.002	ATP binding	0.0036
	positive regulation of growth rate	0.033	carboxylesterase activity	0.012
			ATPase activity, coupled to transmembrane movement of substances	0.016
			carbohydrate binding	0.038
10	lipid metabolic process	0.00073	structural constituent of cuticle	1.18E-06
	positive regulation of programmed cell death	0.0019	triglyceride lipase activity	0.0017
	lipid transport	0.0028	lipid transporter activity	0.0021
	body morphogenesis	0.0047	carbon-oxygen lyase activity	0.0046
	cell adhesion	0.0078	receptor binding	0.015
	proteolysis	0.0083	hydrolase activity, hydrolyzing O-glycosyl compounds	0.015
	potassium ion transport	0.038	voltage-gated potassium channel activity	0.017
			cysteine-type peptidase activity	0.033
11	oviposition	0.014	Glycosphingolipid biosynthesis	0.017
	transmembrane transport	0.02	Glycosaminoglycan degradation	0.034
12	embryonic development ending in birth or egg hatching	4.20E-16		
	cell division	9.20E-10		
	morphogenesis of an epithelium	1.30E-06		
	mitotic spindle organization	4.10E-06		
	embryonic pattern specification	2.30E-05		
	hermaphrodite genitalia development	0.00012		
	cell fate commitment	0.00015		
	gastrulation	0.00042		
	gonad development	0.00055		
	reproduction	0.0072		
	DNA replication	0.014		
	meiotic chromosome segregation	0.024		
	positive regulation of growth	0.026		
	nematode larval development	0.027		
	establishment or maintenance of cell polarity	0.028		
	growth	0.049		
