# Supplementary material for: Comparative toxicogenomic responses of mercuric and methyl-mercury
Source: BMC Genomics. 2013 Oct 11;14:698. doi: 10.1186/1471-2164-14-698 (PMC3870996; doi:10.1186/1471-2164-14-698)
Supplement: Additional file 6: Table S1 — NOAEL, EC20 and EC50 for Mercurials in Human Cells. Table S2. Nucleotide sequences of primers used for qRT-PCR. Table S3. Sequences of siRNA used in mammalian cell culture. [file 1471-2164-14-698-S6.pdf]

SUPPLEMENTAL TABLE 1

NOAEL, EC_{20} and EC_{50} for Mercurials in Human Cells

	SK-N-SH		HepG2		HEK293	
	$HgCl_2$	MeHgCl	$HgCl_2$	MeHgCl	$HgCl_2$	MeHgCl
NOAEL	14 µM	1.7 µM	25 µM	18 µM	4.8 µM	2.2 µM
EC_{20}	22 µM	5.1 µM	50 µM	27 µM	7.7 µM	3.9 µM
EC_{50}	29 µM	9.7 µM	58 µM	33 µM	17 µM	6.8 µM

Supplemental Table 2

Primers Sequences used for qRT-PCR

Gene	Forward (5′→3′)	Reverse (5′→3′)
***C. elegans* Genes**		
mlc-2	TTGACAGGAACTGACCCAGAGG	ATAGCCTTGACCTCATCCTCG
mtl-1	CGCCAAATCTCATCACAAA	CGTGAATGTTGCAAACACCT
mtl-2	CCGAACAATTGAACGGTCAC	CCTGCACAAAGACTTCCTGG
**Mammalian Genes**		
β-actin	GATATCGCTGCGCTGGTCGTC	ACGCAGCTCATTGTAGAAGGTGTGG
ABCG2	AGCTCGAAGGAAAGATCCAA	TCGCCATCACAACATCATCT
BACE1	CCAGAGGCAGCTGTCCAGCAC	GTGACGTTGGGGCCATGGGG
BACE2	CAGCTGGCGTGCTGGACGAA	GGGCTGAATGTAAAGCTGAGGCAGG
CHKA	TGAATTTCAAGGGGCTGAGGCCA	CGCCGGCTCGGGATGAACTG
CHKB	CAGCCCGAGGAGCTGAGGGT	AGGGAGTCCACGCCCTGCAA
ELOVL3	TTCGAGGAGTATTGGGCAAC	AAGATTGCAAGGCAGAAGGA
ELOVL6	GTGCTCTTCGAACTGGTGCT	CCCAGAATTTGCTGACAGGT
GCLC	GTGGATGTGGACACCAGATG	GCGATAAACTCCCTCATCCA
PARG	TTTTGCGAGCAGGAGAAGTT	CAGTTCGCTCACCATTCTCA

SUPPLEMENTAL TABLE 3

Sequences of siRNA used in mammalian cell culture

Gene	Target Sequence
ABCG2	CTGGTCTAATTTATTAATCTA
BACE1	CACAGTGGCACTAGCATTATA
BACE2	TACATGTGCCACCAACATAAA
CHKA	AGCCGGCGATTAGATACTGAA
CHKB	CACGAAGATGGCGCAATTTCA
ELOVL3	ACGGTTCATCATCCTGCGTAA
ELOVL6	TAGGTTGATTTAACCCAGTAA
GCLC	TAGGATCAGTAAATCCCGATA
PARG	CTGGATCACAATGAATGTCTA
